# Antiviral activity of zinc against hepatitis viruses: current status and future prospects

**DOI:** 10.3389/fmicb.2023.1218654

**Published:** 2023-10-16

**Authors:** Shiv Kumar, Shabnam Ansari, Sriram Narayanan, C. T. Ranjith-Kumar, Milan Surjit

**Affiliations:** ^1^Virology Laboratory, Centre for Virus Research, Therapeutics and Vaccines, Translational Health Science and Technology Institute, NCR Biotech Science Cluster, Faridabad, Haryana, India; ^2^University School of Biotechnology, Guru Gobind Singh Indraprastha University, New Delhi, India

**Keywords:** zinc, viral hepatitis, hepatitis A virus, hepatitis B virus, hepatitis C, hepatitis E virus

## Abstract

Viral hepatitis is a major public health concern globally. World health organization aims at eliminating viral hepatitis as a public health threat by 2030. Among the hepatitis causing viruses, hepatitis B and C are primarily transmitted via contaminated blood. Hepatitis A and E, which gets transmitted primarily via the feco-oral route, are the leading cause of acute viral hepatitis. Although vaccines are available against some of these viruses, new cases continue to be reported. There is an urgent need to devise a potent yet economical antiviral strategy against the hepatitis-causing viruses (denoted as hepatitis viruses) for achieving global elimination of viral hepatitis. Although zinc was known to mankind for a long time (since before Christ era), it was identified as an element in 1746 and its importance for human health was discovered in 1963 by the pioneering work of Dr. Ananda S. Prasad. A series of follow up studies involving zinc supplementation as a therapy demonstrated zinc as an essential element for humans, leading to establishment of a recommended dietary allowance (RDA) of 15 milligram zinc [United States RDA for zinc]. Being an essential component of many cellular enzymes and transcription factors, zinc is vital for growth and homeostasis of most living organisms, including human. Importantly, several studies indicate potent antiviral activity of zinc. Multiple studies have demonstrated antiviral activity of zinc against viruses that cause hepatitis. This article provides a comprehensive overview of the findings on antiviral activity of zinc against hepatitis viruses, discusses the mechanisms underlying the antiviral properties of zinc and summarizes the prospects of harnessing the therapeutic benefit of zinc supplementation therapy in reducing the disease burden due to viral hepatitis.

## 1. Introduction

Zinc is the second most abundant trace element found in humans. A healthy adult body contains 2–4 grams of zinc. Zinc is involved in several biological functions including growth, development, maintenance of immune system and disease resistance. It shows broad anti-inflammatory and anti-oxidant properties. At molecular level, many enzymes and proteins that regulate DNA replication, transcription, signal transduction and apoptosis require zinc for their activities (Maret and Sandstead, 2006; John et al., 2010; Ryu et al., 2020).

Plasma or serum zinc levels between 0.8 and 1.20 μg/ml is considered normal in a healthy individual. Zinc is absorbed in the small intestine by a carrier-mediated mechanism in a concentration dependent manner and increases with increasing dietary zinc (Roohani et al., 2013; Ryu et al., 2020). The portal system delivers absorbed zinc directly to the liver, which directs its circulation for delivery to the other tissues. About 70% of the zinc in circulation is bound to albumin, and any condition that alters serum albumin concentration can have a secondary effect on serum zinc levels (Roohani et al., 2013).

Zinc level gets affected by many conditions such as infections, changes in steroid hormone levels, and muscle catabolism during weight loss or illness (Roohani et al., 2013; Ryu et al., 2020). Around 2 billion people are suggested to have prolonged zinc deficiency worldwide, majority of which includes population from economically weaker countries (Prasad, 2013). Zinc deficiency enhances vulnerability to many viral infections and increasing number of studies support the therapeutic benefit of zinc supplementation in alleviating several viral diseases (Sadeghsoltani et al., 2021). This review provides a comprehensive summary of the antiviral effect of zinc in viral hepatitis and discusses the possible scope of better management of viral hepatitis cases using more potent zinc formulations.

## 2. Antiviral effect of zinc on hepatitis viruses

Majority of viral hepatitis is caused by the Hepatitis A virus (HAV), Hepatitis B virus (HBV), Hepatitis C virus (HCV), Hepatitis D virus (HDV) and Hepatitis E virus (HEV; Myers et al., 2002). In addition, Herpes viruses such as Epstein–Barr virus (EBV), Cytomegalo virus (CMV), Adenovirus and Varicella zoster virus (VZV) also induce hepatic injury (Lalazar and Ilan, 2014). Herpes simplex virus (HSV) induced hepatitis is a rare cause of acute liver failure (Chaudhary et al., 2017).

### 2.1. Hepatitis A virus

HAV is a positive stranded nonenveloped RNA virus that is transmitted via the fecal-oral route (Martin and Lemon, 2006; Traore et al., 2012). It is the most common cause of acute viral hepatitis globally (Hauri et al., 2006). It does not cause chronic hepatitis but it adds to further deterioration of liver infected with other hepatotropic viruses (Keeffe, 1995; Vento et al., 1998). A vaccine is available against it (Hauri et al., 2006). No specific treatment is available against it but the disease is self-limiting and there is no lasting injury.

### 2.2. Hepatitis B virus

HBV is transmitted through exposure to contaminated blood products and body fluids (Myers et al., 2002). It is a DNA virus. Chronic infection with hepatitis B virus (HBV) is estimated to affect 400 million individuals globally, and it is the leading cause of HCC (Lok et al., 2001). Vaccines and antiviral therapies are available against HBV (Das et al., 2019; Zhu et al., 2022).

### 2.3. Hepatitis C virus

HCV is transmitted through exposure to contaminated blood products (Myers et al., 2002). HCV affects more than 170 million people worldwide (Bhatia et al., 2014; Manns et al., 2017). Coinfections of hepatitis viruses are frequently observed in clinical setting. Furthermore, their propensity for chronicity sets the stage for superinfection with other viruses (Myers et al., 2002). It frequently causes chronic infection, leading to hepatocellular carcinoma (HCC; Vento et al., 1998; Manns et al., 2017). No vaccine is available against it. Treatment options for HCV cases include a combination of broadly-acting antivirals (such as peg-interferon, ribavirin) and specific direct-acting antiviral (Sofosbuvir; Bhatia et al., 2014).

### 2.4. Hepatitis D virus

HDV is transmitted through exposure to contaminated blood products and body fluids (Myers et al., 2002). It requires the HBV surface antigen (HBsAg) to replicate and is dependent on the latter (Huang and Lo, 2014). Around 5% of HBV carriers (approximately 20 million individuals) are coinfected with the HDV (Myers et al., 2002). No vaccine is available against HDV but HBV vaccinated people are protected from it as it is a significant threat only in HBV infected individuals. Recently, Bulevirtide was shown to be a potential treatment option against HDV (Dietz-Fricke et al., 2023).

### 2.5. Hepatitis E virus

HEV is a positive stranded quasi-enveloped RNA virus (Nimgaonkar et al., 2018). It is transmitted via the fecal-oral route. It can also get transmitted via blood transfusion. Zoonotic transmission of HEV from animals to human is also reported. It is a major cause of acute viral hepatitis globally (Primadharsini et al., 2021). HAV and HEV are major cause of community level outbreaks and epidemics in areas with poor sanitary conditions (Wu et al., 2016; Primadharsini et al., 2021). It may cause chronic infection in immune compromised individuals. At present, a vaccine against HEV is available in China (Wu et al., 2016). HEV cases are self-limiting in otherwise healthy individuals. A combination of broadly-acting antivirals are the option for off-label therapy in severe HEV cases (Netzler et al., 2019).

There is a need to formulate more potent, side-effect free therapeutics for treatment of viral hepatitis cases (Cowan et al., 2011). Controlled zinc supplementation is known to be a safe, side effect free therapy against Wilson’s disease (Brewer et al., 2000). Zinc is widely used as an antimicrobial agent, without any side effect (Wessels et al., 2022). Zinc supplementation is a part of standard care in the treatment of diarrhea in infants (Bajait and Thawani, 2011). Multiple laboratories have independently evaluated the antiviral potential of zinc against hepatitis viruses using diverse experimental approaches. Compilation and careful interpretation of the available data will be useful in evaluating the therapeutic potential of zinc in the treatment of viral hepatitis cases. Below sections compile majority of the available data on antiviral activity of zinc *in vivo* and *in vitro*.

### 2.6. Clinical trials on evaluation of therapeutic benefit of zinc compounds in viral hepatitis patients

Multiple clinical trials have been performed to determine the therapeutic benefit of zinc supplementation in viral hepatitis patients (Table 1). Majority of the trials involved HCV patients (10 trials), one trial involved HBV patients and one trial involved HEV patients. In some studies, serum zinc level was measured in the patients before and after the treatment regimen and compared to that of the placebo group. Pre-existing zinc deficiency was observed in 4 trials while normal zinc level was observed in 3 trials and zinc level was not measured in 5 trials. Zinc supplementation increased the serum zinc level in three of the four zinc deficient groups tested. Effect of zinc supplementation on disease outcome was evaluated by measuring the levels of serum albumin, ALT (alanine aminotransferase), AST (aspartate aminotransferase) and viral load [sustained viral response (SVR)]. In trials involving only zinc supplementation or zinc supplementation in addition to the standard antiviral therapeutics, there was an increased therapeutic response, compared to the placebo group. However, there was lack of positive clinical outcome with Zinc supplementation in four trials of chronic HCV cases (Table 1). In summary, these studies support the therapeutic benefit of zinc supplementation in a subset of viral hepatitis patients.

**Table 1:** Clinical trials on evaluation of therapeutic benefit of zinc compounds in viral hepatitis patients.

Disease etiology	Number of participants (NT_Zn_ vs. NT_placebo_)	Zinc dosage, treatment duration	Effect of zinc supplementation					Effect of Zinc Supplementation	Reference
			Serum Zinc levels Pre/Post (μg/dl; m ± SD)	SVR % (Np/NT)	Serum Albumin Pre/Post (g/dl; m ± SD)	ALT (IU/ml)	AST (IU/ml)		
A. Positive clinical outcome in viral hepatitis patients with zinc supplementation									
(IFN + RBV + Zinc) vs. (IFN + RBV + Placebo)									
HCV	NT_(VitC/E + Polaprezinc)_ = 9 NT_Placebo_ = 12	Polaprezinc: 75 mg/2× day (17 mg zinc), 48 weeks	IGpre/post: 63.7 ± 4.4/68.1 ± 5.3 CGpre/post: 62.9 ± 3.1/62.6 ± 2.7	IG: NR CG: NR	IGpre/post: 3.9 ± 0.1/3.8 ± 0.2 CGpre/post: 3.8 ± 0.2/3.9 ± 0.1	IGpre/post: 47 ± 6/23 ± 3 CGpre/post: 61 ± 6/38 ± 3	IGpre/post: 53 ± 13/30 ± 4 CGpre/post: 50 ± 8/37 ± 5	Polaprezinc induces antioxidative functions in the liver resulting in reduced hepatocyte injury during PEG-IFN α-2b plus ribavirin therapy	Murakami et al. (2007)
(IFN + Zinc) vs. (IFN + Placebo)									
HCV	NT_polaprezinc_ = 15 NT_Zinc_ Sulphate = 9 NT_placebo_ = 10	Polaprezinc: 75 mg/2× day (34 mg zinc) Zinc Sulphate: 150 mg/2× day (34 mg zinc), 20 weeks	IG:NR CG:NR	IG: 9/24 (37.5%) CG: 2/10 (20.0%)	IG: NR CG: NR	IGpost:97 ± 16 CGpost:101 ± 20	NR	Polaprezinc works better than Zinc Sulphate in increasing the therapeutic response of IFN-α against chronic hepatitis C	Nagamine et al. (2000)
HCV	NT_Polaprezinc_ = 35 NT_placebo_ = 40	IFN: 106 units/day Polaprezinc: 75 mg/x2 day (34 mg Zn), 25.7 weeks	IGpost: 75.4 ± 21.3 CGpost: 84.1 ± 19.8	IG: 18/32 (56.3%) CG: 8/36 (22.2%)	IG: NR CG: NR	IGpost:79.0 ± 76.6 CGpost:66.4 ± 31.7	IGpost:76.5 ± 65.9 CGpost 65.9 ± 40.3	Zinc supplementation enhances the response to interferon therapy in patients with intractable chronic HCV infections	Takagi et al. (2001)
(RBV + Zinc) vs. (RBV)									
HEV	NT_(RBV + Zinc Acetate dehydrate)_ = 3 NT_(Zinc Acetate dehydrate)_ = 5	NR	IGpre/post: NR CGpre/post: NR	IG: 2/3 (67%) CG: 0/5 (0%)	IG: NR CG: NR	NR	NR	Zinc supplementation increases serum zinc level and reduces HEV load and AST/ALT levels in HEV patients who do not respond to ribavirin therapy	Horvatits et al. (2023)
(BCAA + Zn) vs. (BCAA + Placebo)									
HBV	NT _(BCAA + ZnSO4)_ = 19 NT_BCAA_ = 21	BCAA: 4 g/day Zinc Sulfate: 200–600 mg/day (variable), 25.7 weeks	IGpre/post: 58.4 ± 9.2/59.7 ± 0.27 CGpre/post: 60.2 ± 9.0/61.1 ± 0.15	IG: NR CG: NR	IGpre/post: 3.3 ± 0.2/2.2 ± 0.07 CGpre/post: 3.3 ± 0.2/2.0 ± 0.08	NR	NR	Zinc supplementation along with branched-chain amino acid improves disorders of nitrogen metabolism in liver cirrhosis in HBV patients	Hayashi et al. (2007)
(Zn) vs. (Placebo) in Viral cirrhosis									
HCV	NT_Polaprezinc_ = 32 NT_placebo_ = 30	Polaprezinc: 1 g/ day, 5 years	IGpre: NR CGpre: NR	IG: 499.6 (8.4–850) CG: 576.0 (7.4–850)	IGpre: NR CGpre: NR	IGpost: 86.3 (41–231) CGpost: 93.39 (45–201)	IGpost: 61 (40–118) CGpost: 82.1 (46–138)	Polaprezinc supplementation reduced AST level, ALT level and incidence of HCC.	Matsuoka et al. (2009)
HCV	NT_Polaprezinc_ = 14 (comparison of parameters pre- and post-zinc treatment)	Polaprezinc: 75 mg/x3 day (51 mg Zinc), 25.7 weeks	IGpre/post: 64 ± 15/78 ± 26	IG: NR	IG: NR	IGpre/post: 106 ± 33/65 ± 23	IGpre/post: 92 ± 33/63 ± 23	Polaprezinc exerts an anti-inflammatory effect on the liver in patients with HCV-related CLD by reducing iron overload	Himoto et al. (2007)
HCV	NT_Zinc Sulphate_ = 9 (comparison of parameters pre- and post-zinc treatment)	Zinc sulfate 200 mg/day (136 mg zinc), 10.7 weeks	IGpre/post: 74.2 ± 12.4/125 ± 25.0	IG: NR	IGpre/post: 33 ± 4.8/34.5 ± 4.4	IGpre/post: 83/61	NR	Zinc supplementation is beneficial in zinc deficient patients with cirrhosis	Bianchi et al. (2000)
B. Lack of positive clinical outcome in viral hepatitis patients with Zinc supplementation									
(IFN + RBV + Zn) vs. (IFN + RBV + Placebo)									
HCV	NT_Zinc gluconate_ = 18 NT_Placebo_ = 20	Zinc Gluconate: 78 mg/5× day (50 mg zinc), 24 weeks	IG Pre:56.9 ± 16.9 CG Pre: 60.6 ± 10.8	IG: 9/18 (50%) CG: 10/20 (50%)	IG pre: 3.6 ± 0.3 CG pre:3.7 ± 0.4	IGpost: 170 ± 145 CGpost: 146 ± 96	IGpost:135 ± 102 CGpost: 96 ± 86	Zinc supplementation may be a complementary therapy in chronic hepatitis C patients to increase the tolerance to IFN-alpha-2a and ribavirin	Ko et al. (2005)
HCV	NT_Polaprezinc_ = 39 NT_placebo_ = 39	Polaprezinc: 75 mg/2× day (17 mg zinc), 24 weeks	IG Pre:73.3 ± 20.3 CG Pre: 69.8 ± 17.2	IG: 13/39(33.3%) CG:13/39(33.3%)	IG: NR CG: NR	IGpost: 95.6 ± 61.1 CGpost:97.4 ± 59.8	IG: NR CG: NR	Polaprezinc did not show any additional therapeutic benefit in HCV patients treated with IFN and ribavirin	Suzuki et al. (2006)
HCV	NT_Zinc gluconate_ = 16 NT_Placebo_ = 16	Zinc Gluconate: 30 mg zinc/day, 24 weeks	IG Pre/post: 75 ± 19/84 ± 28 CG Pre/post: 62.9 ± 3.1/62.6 ± 2.7	IG: 13/16(81.2%) CG:14/16(87.5%)	IG: NR CG: NR	IGpost: 78 ± 52 CGpost: 65 ± 71	IGpost: 85 ± 75 CGpost: 69 ± 59	30 mg/day zinc gluconate did not significantly improve the outcome of treatment in thalassemia patients with chronic hepatitis C	Abbasinazari et al. (2014)
HCV	NT_Polaprezinc_ = 16 NT_placebo_ = 16	polaprezinc:75 mg (17 mg zinc)x2 day, 48 weeks	IG Post:69.4 ± 6.5 CG Post: 73.9 ± 7.5	IG: 8/16(50%) CG:7/16(43.8%)	IG: NR CG: NR	IG: NR CG: NR	IGpost: 45.6 ± 39.3 CGpost: 48.2 ± 26.9	Polaprezinc did not further improve hematologic side effects, liver function in chronic HCV patients treated with PEG-IFN-α2b and ribavirin.	Kim et al. (2008)

Serum zinc levels are decreased in HCV patients and the underlying mechanism is proposed to be due to the requirement of zinc binding by the viral non-structural proteins NS3 and NS5A (Love et al., 1996; Stempniak et al., 1997; Tellinghuisen et al., 2004). Comparison of serum zinc levels in chronic hepatitis C patients before and after treatment with Direct acting antivirals (DAAs) revealed an increase in the serum zinc level after DAA treatment (Suda et al., 2019). Importantly, they showed that the increased zinc level was not attributed to an increase in the albumin level, but it was a direct outcome of the viral RNA clearance (Suda et al., 2019).

Chronic hepatitis due to HCV infection is a known risk factor of HCC. In an interesting study, Hosui et al. evaluated the effect of oral zinc supplementation on the risk of HCC development in DAA treatment-cured chronic hepatitis C suffering individuals (Hosui et al., 2021). One year and three year follow up study after the end of DAA therapy showed cumulative incidence rates of 1.8 and 5.6%, respectively in the no zinc supplemented (control) group. None from the zinc supplemented group developed HCC. Moreover, serum zinc concentration was significantly higher in the no HCC group than the HCC group (Hosui et al., 2021). These data suggest the therapeutic benefit of zinc supplementation in reducing the risk of HCC development in individuals recovered from chronic hepatitis C.

In addition to viral infections, there are other inducers of hepatic dysfunction such as alcohol consumption. Multiple independent clinical trials have been carried out to assess the therapeutic benefit of zinc supplementation in non-viral hepatitis patients with liver cirrhosis (Overbeck et al., 2008; Diglio et al., 2020). Zinc supplementation significantly increased the serum zinc level, reduced the serum albumin level and improved the overall disease condition in those patients, further attesting the therapeutic benefit of zinc in hepatitis patients (Overbeck et al., 2008; Diglio et al., 2020).

### 2.7. Antiviral effect of zinc compounds in cell culture-based infection/replicon models of hepatitis viruses.

Several independent studies support the antiviral role of zinc on replication and survival of HAV, HCV and HEV (Table 2; Figure 1). All studies used safe dose of zinc, which did not affect the viability of the cells that were used in the experiment. Zinc sulphate partially inhibited the replication of HAV in Huh7 (human hepatoma) cells (Ogawa et al., 2019). Zinc sulphate and zinc chloride inhibit replication of the genomic length HCV RNA at a concentration of 100 μM, with maximum effect at 48 h of treatment (Yuasa et al., 2006). Another study by Gupta et al. compared the HCV inhibitory effect of zinc oxide nanoparticles [ZnO(NP)] and tetrapods [ZnO(TP)] with conventional zinc salts such as ZnSO_4_, which revealed the superior antiviral potency of the ZnO(TP) against HCV (Gupta et al., 2022). Zinc salts, ZnO(NP) and ZnO(TP) also show antiviral activity against HEV, latter being the most potent (Gupta et al., 2022). ZnO(NP) and ZnO(TP) are nanoparticle conjugated variants of ZnO, which is better absorbed in the intestine, possess better bioavailability and reduced undesirable side effect characteristics (Sirelkhatim et al., 2015; Jiang et al., 2018). Therefore, ZnO(NP) and ZnO(TP) are safer alternatives to the conventional zinc salts for therapeutic use. Inhibitory effect of ZnO(TP) was comparable to that of sofosbuvir, a well-known DAA used in the treatment of HCV cases, further testifying the antiviral potential of ZnO(TP) against HCV (Yuasa et al., 2006; Gupta et al., 2022).

**Table 2:** Anti-viral effect of zinc on hepatitis viruses in respective cell culture-based infection models/replicon models.

Disease Etiology	Model system	Zinc species and dosage (μM)	Percentage reduction in viral RNA level	Affected stage of the viral life cycle	Reduction in viral load	Reference
HAV	Huh7, infectious HAV	Zinc Sulphate: 100	55	Replication	Yes	Ogawa et al. (2019)
HCV	Huh7, HCV replicon	Zinc Sulphate: 100 Zinc Chloride: 100	60	Replication	Yes	Yuasa et al. (2006)
HCV	Huh7 cells, HCV replicon	Zinc Oxide (NP*): 200 Zinc Oxide (TP*): 200 Zinc Sulphate: 200	90	Replication	Yes	Gupta et al. (2022)
HEV	Huh7 cells, infectious HEV	Zinc Sulphate: 200 Zinc Acetate: 200	90	Replication	Yes	Kaushik et al. (2017)
HEV	Huh 7.5, HEV replicon	Zinc salt^#^: 115	95	Replication	Yes	Horvatits et al. (2023)
HEV	Huh7, HEV replicon	Zinc Oxide (NP*): 100 Zinc Oxide (TP*): 100 Zinc Sulphate: 100	90	Replication	Yes	Gupta et al. (2022)

^*^NP, Nanoparticle; TP, Tetrapod.

# Full name not reported.

**Figure 1:**
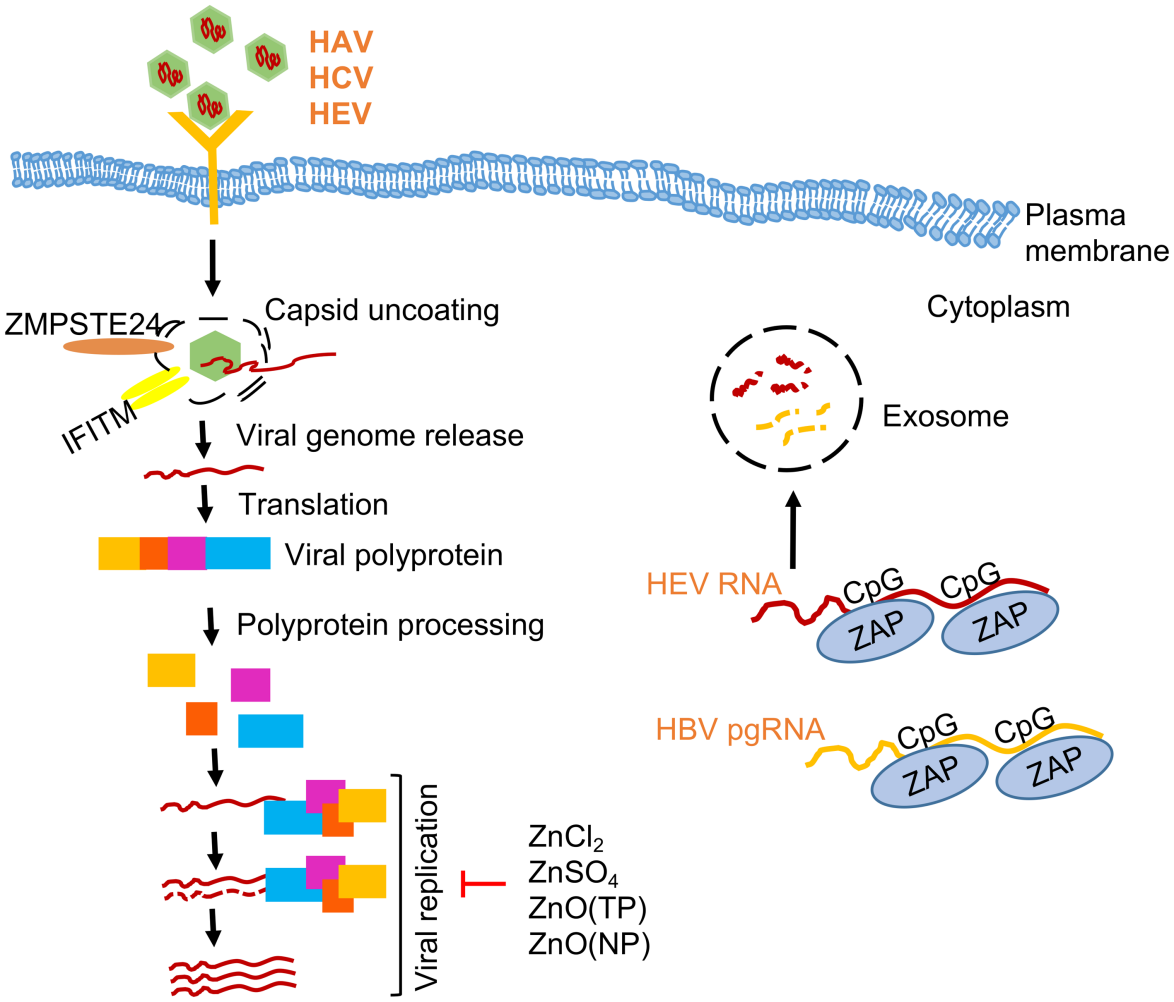
Effect of zinc on hepatitis viruses. A simplified scheme of life cycle of HAV, HCV and HEV is shown. Note that HAV and HEV are quasi-enveloped whereas HCV is an enveloped virus. Zinc salts (ZnCl_2_, ZnSO_4_), ZnO nanoparticles (NP) and ZnO tetrapod-shaped nanoparticles (TP) inhibits the replication of Hepatitis A (HAV), Hepatitis C (HCV) and Hepatitis E viruses (HEV). Zinc finger antiviral protein (ZAP) binds to CpG motifs in HEV RNA and HBV pre-genomic (pg) RNA and targets them for degradation.

## 3. Broad-spectrum antiviral effect of zinc: insight from studies on other viruses

**Figure 2:**
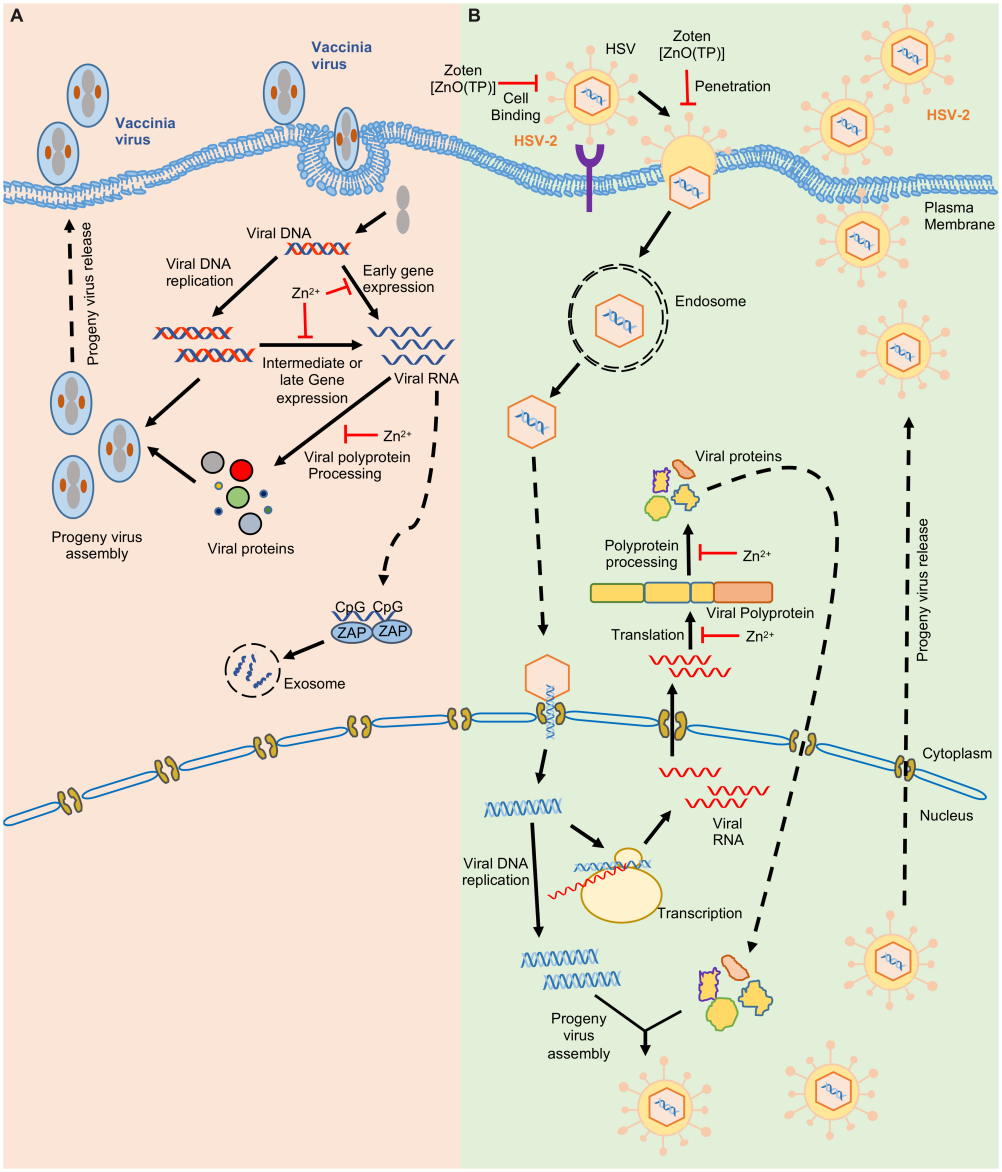
Effect of zinc on Vaccinia and Herpes simplex virus-2. Schematic showing the life cycle of Vaccinia virus **(A)** Herpes Simplex virus-2 **(B)**. ‘’ indicates the steps inhibited by zinc.

**Figure 3:**
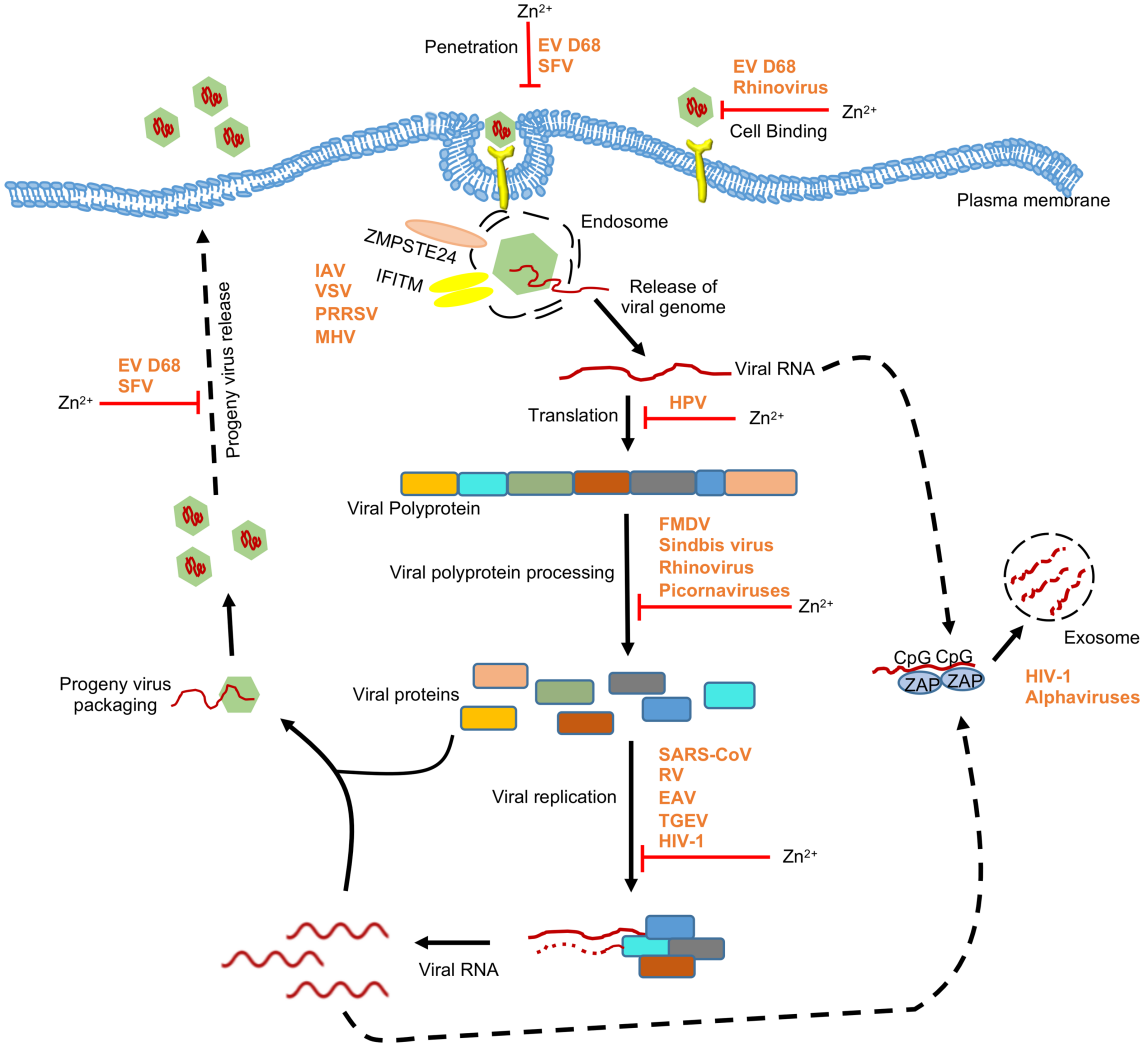
Effect of zinc on RNA viruses. Schematic showing the effect of zinc on life cycle of RNA viruses. Apart from directly inhibiting different stages of viral life cycle, zinc mediates its antiviral activity through zinc-containing proteins such as ZAP and ZMPSTE24. ZAP binds to CpG motif in viral RNA and targets them for exosomal degradation. ZMPSTE24 and IFITM complex interferes with entry of viruses. EV D68, enterovirus D68; HPV, human papilloma virus; RSV, respiratory syncytial virus; TGEV, transmissible gastroenteritis virus; SFV, Semliki forest virus; SARS-CoV, severe acute respiratory syndrome coronavirus; FMDV, foot and mouth disease virus; HIV-1, human immunodeficiency virus-1; IAV, influenza A virus; VSV, vesicular stomatitis virus; PRRSV, porcien reproductive and respiratory syndrome virus; MHV, mouse hepatitis virus. ‘’ indicates the steps inhibited by zinc.

Antiviral effect of zinc have been demonstrated *in vivo* and *in vitro* in several viruses, including corona viruses, picornaviruses, papilloma viruses, metapneumoviruses, rhinoviruses, herpes simplex viruses, varicella-zoster viruses, respiratory syncytial viruses, retroviruses, SARS-CoV and SARS-CoV-2 etc. (Bracha and Schlesinger, 1976; Gupta and Rapp, 1976; Polatnick and Bachrach, 1978; Katz and Margalith, 1981; Kümel et al., 1990; Hulisz, 2004; Krenn et al., 2009; Te Velthuis et al., 2010; Liu and Kielian, 2012; Wei et al., 2012; Antoine et al., 2016; Liu et al., 2021; Samad et al., 2021). Based on the available literature, mechanism underlying the antiviral properties of zinc may be broadly classified into two categories: (a) direct inhibitory effect on the different stages of the life cycle of the virus and (b) indirect effect of zinc attributed to its ability to modulate various host cellular processes and immune response. Zinc shows direct inhibitory action against several viruses. It acts by interfering with different steps of the viral life cycle, it inhibits the activity of key viral proteins and competes with other bivalent ions such as manganese, magnesium or calcium to interrupt the function of viral proteins. Direct antiviral activity of zinc against viruses is schematically illustrated in Figures 2, 3.

### 3.1. Effect of zinc on infectivity of the virus and target cell entry

Zinc may directly accumulate on the virus and inactivate it or interfere with its entry into the target cell. Zinc application leads to its deposition on HSV thereby inactivating the virus and inhibiting its cellular entry (Kümel et al., 1990). Recently zinc Oxide tetrapod nanoparticles with engineered oxygen vacancies (Zoten) were shown to possess potent therapeutic benefit in HSV-2 (Herpes simplex virus-2) mediated genital herpes (Antoine et al., 2016). Zoten blocks cellular entry of HSV-2 by efficiently trapping and inactivating the virus, thereby preventing the disease (Figure 2). It also enhances T cell and antibody mediated immunity in mice, have adjuvant like properties and thus reduces chances of reinfection (Antoine et al., 2016; Samad et al., 2021). Zinc treatment was shown to moderately inhibit enterovirus D68 attachment and entry into target cells (Figure 3; Liu et al., 2021). In the case of Rhinovirus, zinc may act as a competitive inhibitor of virus binding to the ICAM1 (intercellular adhesion molecule 1) on the host cell surface, which is the receptor for virus entry (Hulisz, 2004; Figure 3). Zinc and Nickel inhibit membrane fusion of SFV (Semliki forest virus) by targeting the viral transmembrane E1 protein (Figure 3; Liu and Kielian, 2012).

### 3.2. Effect of zinc on viral protein translation and polyprotein processing

Zinc inhibits proteolytic processing of nonstructural polyproteins of several viruses such as rhinovirus and Picornavirus (Krenn et al., 2009). Zinc ionophores such as Pyrithione and Hinokitol also demonstrate antiviral activity by inhibiting the processing of picornavirus nonstructural polyprotein (Krenn et al., 2009). Zinc treatment inhibits HSV-2, Sindbis, FMDV (Foot and mouth disease virus) and Vaccinia virus growth in infected cells by blocking their polypeptide processing (Figures 2, 3; Bracha and Schlesinger, 1976; Gupta and Rapp, 1976; Polatnick and Bachrach, 1978; Katz and Margalith, 1981).

### 3.3. Effect of zinc on viral replication and transcription

Replication of viral genome is an essential step for proliferation and maintenance of genomic integrity of the virus. RNA dependent RNA polymerase (RdRp) produced by proteolytic processing of the viral nonstructural proteins plays the central role in the viral replication process. Zinc inhibits RdRp activity of many viruses, including TGEV (Transmissible gastroenteritis virus), SARS-CoV (Severe acute respiratory syndrome coronavirus), EAV (Equine arteritis virus), Rhinovirus and HEV (Hepatitis E virus; Korant et al., 1974; Hung et al., 2002; Te Velthuis et al., 2010; Wei et al., 2012; Kaushik et al., 2017). Different steps in the replication process have been shown to be targeted by zinc for inhibiting RdRp activity. In case of SARS-CoV, zinc treatment reduced template binding and elongation by the RdRp whereas in case of EAV, initiation step of RNA synthesis was inhibited (Te Velthuis et al., 2010). Zinc also inhibits Rhinovirus RdRp activity *in vitro* although the mechanism remains to be understood (Korant et al., 1974; Hung et al., 2002). Clinical trials have shown the therapeutic benefit of zinc in alleviating rhinovirus induced common cold symptoms (Eby et al., 1984; Hulisz, 2004; Kurugöl et al., 2006). Zinc inhibits HIV-1 (Human immunodeficiency virus-1) protease and reverse transcriptase activity (Zhang et al., 1991; Haraguchi et al., 1999; Fenstermacher and DeStefano, 2011). Some other HIV-1 encoded proteins are dependent on zinc to carry out their function (Zheng et al., 1996). Effect of zinc supplementation in HIV infected patients have been investigated in clinical trials (Mocchegiani and Muzzioli, 2000; Bobat et al., 2005; Baum et al., 2010). HIV infected children showed a significant decrease in the frequency of watery diarrhea after 3 months of zinc supplementation. However, neither viral load was altered nor CD4^+^ T lymphocytes level was improved (Bobat et al., 2005). Similar effect of zinc supplementation was observed in HIV infected patients having pneumocystis carinii and candida (Mocchegiani et al., 1995). Another study has shown potent antiviral activity of PEGylated ZnO nanoparticle against H1N1 influenza virus (Ghaffari et al., 2019). Recently, polyamide fibers with embedded zinc ions (zinc oxide) were shown to prevent and deactivate Influenza A virus H1N1 and SARS-CoV-2 (Gopal et al., 2021). Further, higher zinc intake was found to reduce the severity of disease in COVID-19 patients (Asoudeh et al., 2023). In addition, many clinical trials have attempted to evaluate the therapeutic benefit of zinc in COVID-19 patients (Carlucci et al., 2020; Chinni et al., 2021; Gordon and Hardigan, 2021; Thomas et al., 2021; Beran et al., 2022; Tabatabaeizadeh, 2022). Though some studies reported a positive outcome, further investigation is warranted to draw a clear conclusion.

Zinc is also reported to act by inhibiting viral particle production and inhibit viral topoisomerase activity in vaccinia virus, inhibit endosomal membrane fusion in Semliki Forest virus and inhibit viral protein E6 and E7 synthesis (thereby stimulating apoptosis) in Human papilloma virus infected cells (Read et al., 2019).

## 4. Antiviral function of zinc-containing host proteins

Zinc finger antiviral protein (ZAP) is a well characterized host protein that recognizes the CpG dinucleotide present in RNA and targets them for degradation through exosome (Guo et al., 2007; Gonçalves-Carneiro et al., 2022). Since CpG containing RNA is not produced in human, ZAP efficiently targets viral RNA, justifying its antiviral property. Human ZAP contains four zinc finger motifs, located at the N-terminus. Zinc finger motifs mediate its interaction with CpG RNA and mutation of some cysteine residues in the zinc finger motif of ZAP result in loss of its antiviral activity (Guo et al., 2004). Structural studies have clearly demonstrated the role of zinc finger motif of ZAP in mediating its antiviral function (Chen et al., 2012). ZAP also associates with triphosphate motif-containing protein 25 (TRIM25, an E3 ubiquitin ligase), which acts as a co-factor of ZAP and supports its antiviral function (Zheng et al., 2017). P72 RNA helicase (a DEAD box family RNA helicase) also associates with ZAP and helps in its antiviral function (Chen et al., 2008).

ZAP has been shown to inhibit HIV-I by targeting multiple viral mRNA for degradation (Zhu et al., 2011). ZAP inhibits alphaviruses by targeting the CpG dinucleotides in the NSP2 region containing RNA (Nguyen et al., 2023). ZAP inhibits human cytomegalovirus by targeting its UL4/UL5 transcripts (Gonzalez-Perez et al., 2021). In a recent study, Yu et al. demonstrated that expression of ZAP and IFN-β was significantly reduced upon HEV infection (Yu et al., 2021). ZAP was shown to interact with the 5’UTR region of the HEV genome. Knockdown of ZAP decreased phosphorylation of IRF3, thus limiting host innate immune system, while poly(I:C) induction in cells upregulated IRF3 phosphorylation and ZAP, thus inhibiting HEV replication (Yu et al., 2021). Hence ZAP shows antiviral activity against HEV. ZAP also shows antiviral activity against HBV by interacting with the HBV pgRNA (pre-genomic RNA) and targeting it for degradation (Mao et al., 2013).

Considering the pattern and specificity of ZAP binding to CpG RNA, it is expected that ZAP acts as a broad spectrum antiviral factor that acts by targeting the virus while retaining resistance to evolving mutations in the viral genome. Unless the virus encodes a specific mechanism to antagonize CpG RNA binding property of ZAP or deplete ZAP or its cofactors, it is unlikely to escape the antiviral activity of the ZAP. On top of that ZAP is also reported to stimulate the RIG-I signaling pathway, which is a major antiviral response mechanism of the host (Hayakawa et al., 2011).

ZMPSTE24 is another host zinc finger motif containing protein, which shows antiviral activity against many enveloped viruses, including influenza virus, Vesicular stomatitis virus (VSV), Vaccinia virus, Porcine reproductive and respiratory syndrome virus (PRRSV) and arenaviruses (Fu et al., 2017; Katwal et al., 2022; Stott-Marshall and Foster, 2022). A recent report demonstrates that ZMPSTE24 inhibits infection of SARS-CoV-2-spike pseudotyped lentivirus, suggesting its antiviral function against the SARS-CoV-2. A similar phenomenon was also observed in the case of the mouse hepatitis virus (MHV; Shilagardi et al., 2022). ZMPSTE24 acts by interacting with interferon-inducible membrane proteins (IFITM) and preventing fusion of the viral envelope (Fu et al., 2017; Li et al., 2017).

## 5. Effect of zinc on host

### 5.1. Maintenance of zinc homeostasis by metallothioneins and zinc transporters

**Figure 4:**
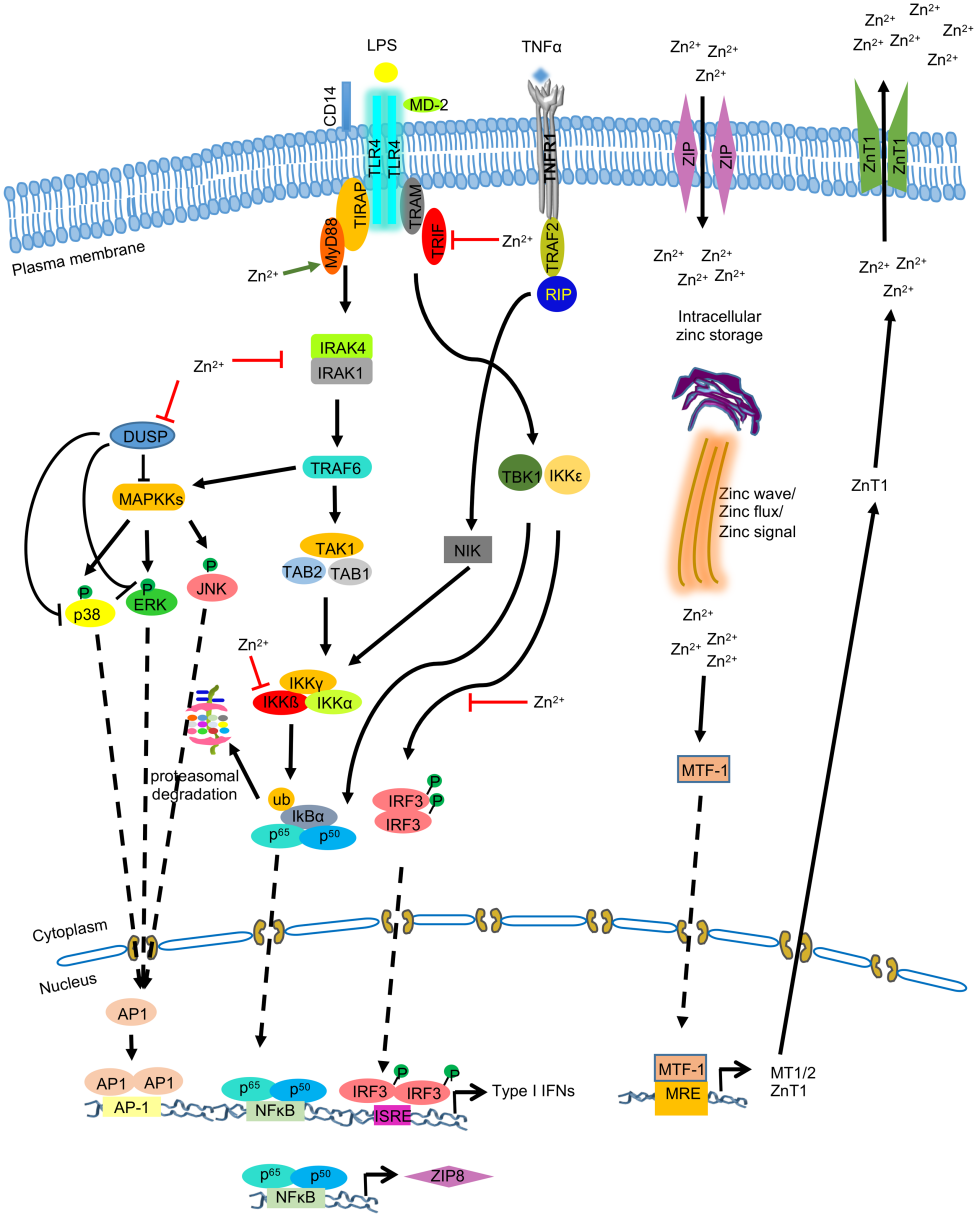
Zinc-Metallothionein homeostasis and zinc signaling in monocytes, macrophages and dendritic cells. LPS mediated stimulation of TLR4 leads to activation of NFκB, IRF3, and MAPK signaling pathways, resulting in production of type I IFNs and ZIP8. Zinc mobilization via ZIP8 increases intracellular zinc level, which acts on MAPK, NFκB, and IRF3 signaling pathways by inhibiting dual specificity phosphatase (DUSP) and by inhibiting IKKβ and IRF3 phosphorylation. Metal Responsive element-binding transcription factor-1 (MTF-1) promotes the synthesis of metallothioneins (MT), which in turn binds to and translocates zinc into organelles. MTF-1 also activates the synthesis of ZnT1, which exports zinc out of the cell. ‘’ indicates the steps inhibited by zinc.

Metallothionein (MT) is a cysteine rich low molecular weight protein, which binds to zinc and copper to regulate their homeostasis in cells and also sequester heavy metals such as cadmium and mercury to alleviate heavy metal poisoning and superoxide stress. There are four MT isoforms in mice (MT1-4) and several isoform/variants in human (Vašák, 2005). MT1 and MT2 are expressed in all organs, while MT3 is expressed in brain and MT4 in stratified tissues. About 10% of human genome encode zinc binding proteins which play crucial biological functions. The availability of zinc is regulated by MT and zinc transporters. MTs sense the intracellular zinc level and modulate zinc through sequestration, distribution and release. Promoter region for MT1 and MT2 contains several metal and glucocorticoid regulatory elements (MREs and GREs). Metal responsive transcription factor 1 (MTF-1) regulates the transcription of MTs (Figure 4; Vašák, 2005). MTF-1 contains six zinc fingers which is responsible for DNA binding, and thus binds to the promoter proximal MREs. Increased Zinc concentration mediate efficient DNA binding of MTF1 (Grzywacz et al., 2015). Another study has shown that during cellular stress, Nitric Oxide (NO) produced by immune cells induce MT1 and MT2 to release zinc and these free zinc ions bind to MTF1, leading to its activation (Stitt et al., 2006). MTs mobilize zinc to nucleus, cytoplasm, golgi and endoplasmic reticulum. MTs also interact with proteins such as GTP, ATP, Gluthatione and these interactions enable their localization in extracellular milieu (Maret, 1994; Jiang et al., 1998; Subramanian Vignesh and Deepe, 2017).

It has been postulated that MTs deliver zinc to the thymulin, which is important for the function of the latter and that aberrant MT regulation can have direct effect on downstream functions of thymulin such as T cell selection, differentiation and function (Subramanian Vignesh and Deepe, 2017). Zinc also regulates the expression of MHC II on dendritic cell (DC) surface. Excess zinc reduces MHC II expression while zinc deficiency elevates MHC II expression on DCs. Thus, it has been proposed that MT-Zn sequestration regulates MHC II expression in DCs, further influencing thymic T cell selection (Kitamura et al., 2006; George et al., 2016).

Several studies also suggest that MT-Zn homeostasis regulates immunological function of bone marrow. Reports have shown that either deficiency of dietary zinc and chronic zinc exposure leads to B-cell and T-cell apoptosis (King et al., 1995; Fraker and King, 2004). Thus, absence of MT expression in bone marrow diminishes zinc homeostasis unless compensatory zinc transporters and MTs are provided. MTs transfer zinc to other metalloproteins including the zinc-dependent transcription factor which regulates the differentiation of precursor cells in bone marrow. For example, Early growth response-1 (Egr-1), a zinc-dependent transcription factor promotes differentiation of monocyte into macrophage (Krishnaraju et al., 1995), while another zinc dependent transcription factor growth factor independent-1 (Gif-1) antagonizes monocyte to macrophage differentiation and rather promotes neutrophil differentiation (Hock et al., 2003). Studies have also shown that exogenously added Zn-MT binds to unknown MT receptor on T-Cell membrane and reduces surface thiol expression. This enhances IL-2 release which promotes T-Cell survival and proliferation (Subramanian Vignesh and Deepe Jr., 2017).

Dendritic cells express MTs in response to thermal stress, which then mediate zinc distribution to regulate intracellular redox environment in DCs. MT1 expressed in DCs induces tolerogenic potential of DCs by promoting differentiation of naïve T cell into FOXP3 expressing Treg cells (Reis e Sousa, 2006; Maldonado and von Andrian, 2010). During inflammation and changed redox state of cells, MTs release zinc. These free zinc ions activate MTs, stimulate MTF-1 and downregulates pro-inflammatory cytokines such as IL6, TNF-α, interleukin IL-1 and also suppresses transcription factor NF-kB. NF-kB induces the expression of pro-inflammatory cytokines 1 L-1, IL-6, TNF- α, which activates MTF-1 transcription factor. MTF-1 upregulates the expression of MTs, and zinc efflux transporter ZnT-1, which helps in maintaining Zn-MT homeostasis and helps recover cells from redox state (Figure 4; Grzywacz et al., 2015).

**Figure 5:**
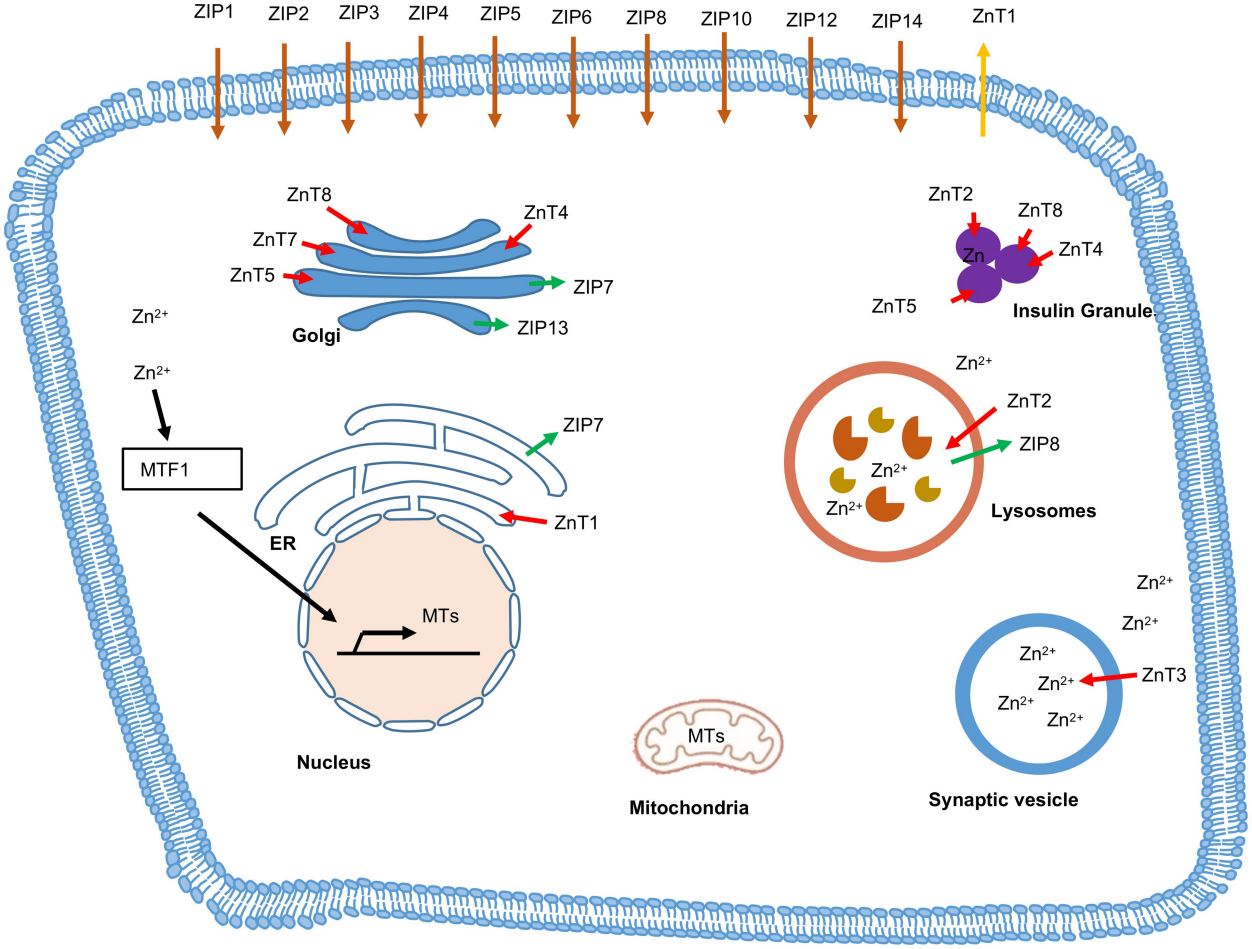
Subcellular localization of Zinc transporters (ZnTs), Zrt- and Irt- like proteins (ZIPs) and Metallothionein (MT). Red arrow indicates the flow of Zn^2+^ from cytosol to cellular organelles via ZnTs and green arrow indicates the flow of Zn^2+^ from cellular organelles to cytosol via ZIPs. Brown arrow indicates the ZIPs located on plasma membrane, which transports zinc inside the cytosol. Orange arrow indicates the ZnTs located on plasma membrane, which transports zinc outside the cell. MTs translocate zinc into nucleus, golgi, endoplasmic reticulum (ER), mitochondria and lysosomes.

Zinc transporters play important roles in Zn^2+^ transport, distribution and homeostasis (Lazarczyk and Favre, 2008; Lichten and Cousins, 2009). Zinc transporters belong to two families: 10 SLC30s/ZnTs and 14 SLC39s/ZIPs. ZIPs mediate influx of Zn^2+^ from extracellular space to intracellular cytoplasm via diffusion, symporter or secondary active transporter. ZnTs export Zn^2+^ from cytoplasmic space to extracellular space (Andrews et al., 2004; Lichten and Cousins, 2009). The ZnTs and the ZIPs with their expression and distribution in the different tissues are listed in the Table 3. Inside the cell, specific set of ZnTs and ZIPs are involved in storage of Zn^2+^ in organelles or its release into cytosol, depending on the state of the cell. Intracellular distribution of ZnTs and ZIPs is schematically shown in Figure 5.

**Table 3:** Expression and distribution of ZnTs and ZIPs.

Name of the protein	Expression and tissue distribution	Reference
A. Expression and distribution of ZnTs		
ZnT1	Ubiquitous	Andrews et al. (2004)
ZnT2	Widely distributed	Itsumura et al. (2013)
ZnT3	Brain	Hildebrand et al. (2015)
ZnT4	Ubiquitous	Huang and Gitschier (1997)
ZnT5	Ubiquitous	Inoue et al. (2002)
ZnT6	Widely distributed	Huang et al. (2007)
ZnT7	Widely distributed	Huang et al. (2007)
ZnT8	Pancreas	Wenzlau et al. (2007)
ZnT9	ubiquitous	Myers et al. (2012)
ZnT10	Small intestine, Liver, Brain	Bosomworth et al. (2012)
B. Expression and distribution of ZIPs		
ZIP1	Ubiquitous	Dufner-Beattie et al. (2006)
ZIP2	Liver, ovary, skin, dendritic cell	Peters et al. (2007)
ZIP3	Widely distributed	Dufner-Beattie et al. (2006)
ZIP4	Small intestine	Andrews (2008)
ZIP5	Small intestine, kidney, pancreas	Guo et al. (2014)
ZIP6	Widely distributed	Mathews et al. (2006)
ZIP7	Widely distributed, Colon	Groth et al. (2013)
ZIP 8	Widely distributed	Li et al. (2016)
ZIP9	Widely distributed	Hara et al. (2017)
ZIP 10	Widely distributed, Renal cell	Hara et al. (2017)
ZIP 13	Hard and connective tissues	Hara et al. (2017)
ZIP14	Widely distributed	Hara et al. (2017)

Through sequence analysis it was observed that the ZIP family of transporters contains 8 transmembrane domains (TMDs-1-8). These 8 transmembrane domains are responsible for the zinc transport. A cytoplasmic segment lies between the 3^rd^ and 4^th^ TMD (Taylor and Nicholson, 2003; Bin et al., 2011). These proteins are further divided into four subfamilies- I, II, gufA, and LIV-1 (Taylor and Nicholson, 2003). Amongst these, the LIV-1 sub family members contain a HEXXH motif within TM5 (transmembrane domain 5) and varying lengths of the N-terminal extracellular domain (ECD; Taylor and Nicholson, 2003). Out of the 14 ZIPs identified in mammals, ZIP1, ZIP2, ZIP3 belong to the ZIP II subfamily. ZIP 9 is a member of the ZIP I subfamily and ZIP 11 is of the gufA subfamily (Yu et al., 2013). The remaining 9 ZIPs belong to LIV-1 subfamily (Ryu et al., 2020). The functional roles of LIV-1 subfamily proteins are predominantly due to their ECDs. The role of the ZIP-CTD present between TM3 and TM4 remains unknown (Bin et al., 2018).

The transport mechanisms being used by ZIPs is not yet clearly elucidated and requires further study, but the cell-based assays done using isotopes suggest that ZIP2 controls the zinc flux through a time, temperature and concentration dependent manner as seen in erythroleukemia cells (Gaither and Eide, 2000). It was further seen that ZIP2 was stimulated only by HCO_3_-, with a high affinity to zinc, suggesting a Zinc- HCO_3_- symporter mechanism (Gaither and Eide, 2000). ZIP8 has also been shown to act as a Zn- HCO_3_^−^ symporter in complementary RNA injected Xenopus oocytes (Liu et al., 2008).

ZnTs are part of a superfamily of cation diffusion facilitators. Mammalian ZnTs are predicted to have at least 6 TMDs (Fukada and Kambe, 2011). The ZnTs possess a histidine/serine rich loop of differing lengths between the 4th and 5th TMDs (Fukada and Kambe, 2011). Further ZnTs contain a large cytoplasmic domain at the C- terminus (CTD) with a copper chaperone like architecture (Lu and Fu, 2007). This domain has an important role in diabetes research as mutations in the CTD of ZnT8 increases the risk of developing diabetes (Parsons et al., 2018). The transport mechanism utilized by ZnTs is not clearly understood in the mammalian system, but studies done on *E. coli* Zinc transporter YiiP suggests that ZnTs control the zinc efflux through a Zn^2+^/H^+^ antiporter (Lu and Fu, 2007).

Zinc is an essential component of several cellular processes in the host. It is also essential for normal development and functioning of the innate and adaptive immune systems. The diverse functional properties help in further strengthening the antiviral action of zinc, either by stimulating a better immune response in the host and/or by promoting the synthesis/activation of antiviral factors/pathways, as described in the following sections.

### 5.2. Modulation of host immune system by zinc

#### 5.2.1. Zinc signaling in monocytes, macrophages, and dendritic cells

There is a reduction in phagocytosis of macrophages, decrease in chemotaxis of polymorphonuclear cells and decrease in the production of proinflammatory cytokines upon zinc deficiency (Rink and Kirchner, 2000; Bonaventura et al., 2015). Upon entering the host, pathogens are recognized by the pattern recognitions receptors (PRRs) such as Toll-like receptors (TLRs), which initiate different signaling cascades, leading to production of host factors essential for survival.

Except TLR3, activation of all other TLRs by various ligands increases intracellular Zn^2+^ level, which inhibits the phosphorylation of IRF3 in murine macrophages, leading to reduced production of type I interferons such as IFNβ. Conversely, zinc deficiency increases the level of LPS-induced IFNβ (Haase et al., 2008; Brieger et al., 2013; Figure 4). Thus, increased Zn^2+^ level negatively regulates TRIF/TRAM-dependent signaling pathway (Figure 4). Increased Zn^2+^ level also activates MAP kinases (mitogen activated protein kinases) in LPS-treated macrophages in a IRAK-TRAF-dependent manner, leading to production of proinflammatory cytokines. Zinc mediated inhibition of MAPK phosphatases such as the dual-specificity phosphatases (DUSPs) and degradation of IRAK1 is proposed to control the effect of zinc on MAPKs (Haase et al., 2008; Wan et al., 2014; Figure 4). Further, zinc modulates MyD88-dependent activity of the transcription factor NFκB. Hasse et al. reported that zinc depeletion reduced LPS-induced phosphorylation of IKKβ in monocytes and reduced DNA binding by NFκB (Haase et al., 2008). However, few other studies reported inhibition of NFκB activity by zinc (Von Bülow et al., 2007; Haase and Rink, 2009; Prasad et al., 2011). Subsequently, it was found that zinc transporter ZIP8 was a target of NFκB (Liu et al., 2013). NFκB mediated upregulation of ZIP8 levels further increases intracellular Zn^2+^ level, which in turn inhibits IKKβ phosphorylation, leading to the inhibition of NFκB activity. Zinc also inhibits the activity of phosphodiesterase (PDE) in monocytes, leading to the inhibition of NFκB activity (Von Bülow et al., 2007).

#### 5.2.2. Zinc signaling in T cells

**Figure 6:**
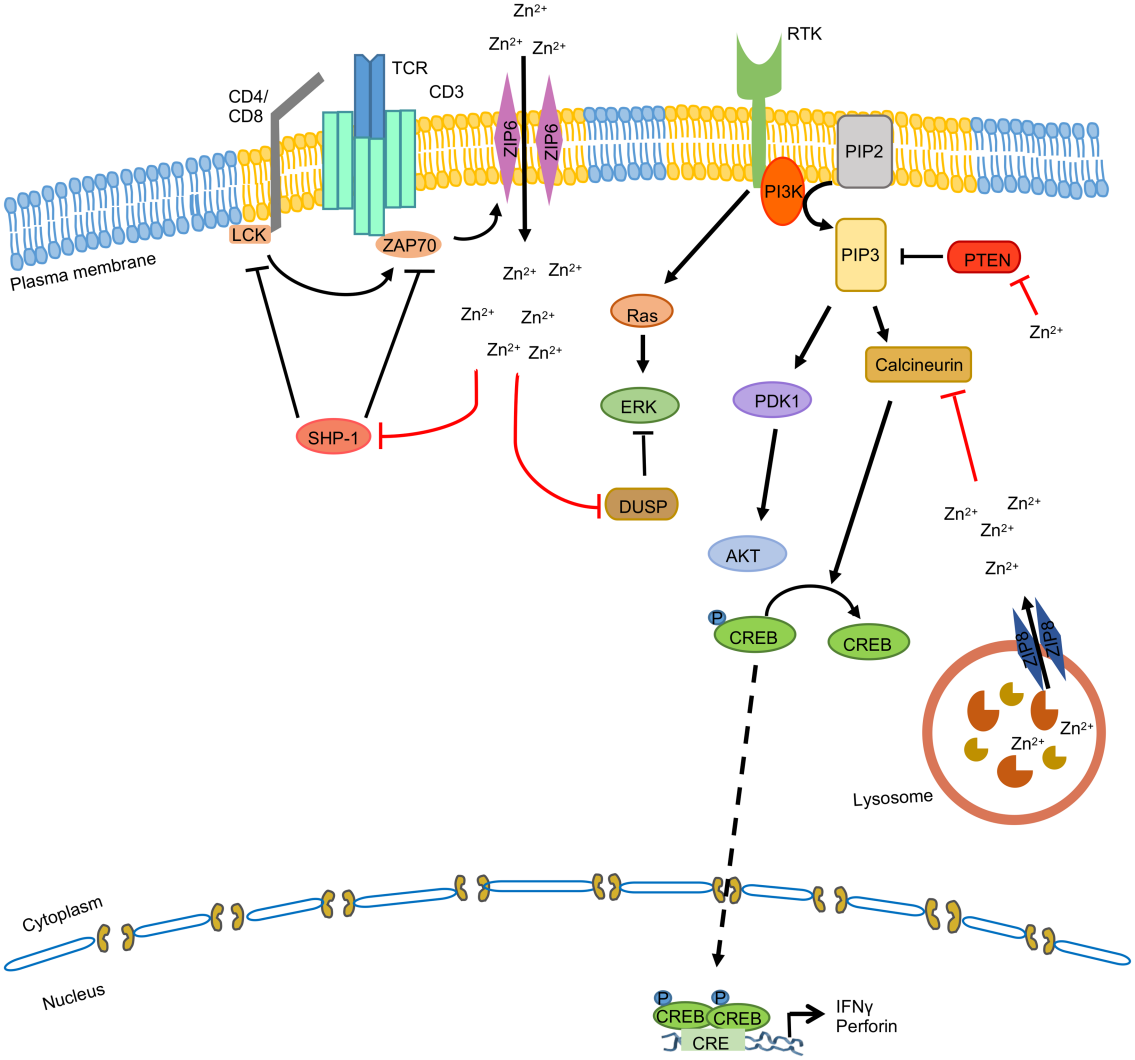
Zinc signaling in T cells. TCR stimulation induces ZAP70 mediated phosphorylation of ZIP6, leading to its localization to the immunological synapse in lipid rafts (yellow). ZIP6 mediates Zn^2+^ influx, which inhibits SHP-1 mediated dephosphorylation of the LCK and DUSP mediated dephosphorylation of the MAPKs. ZIP8 is present on the lysosome and it mobilizes lysosomal Zn^2+^ to cytoplasm, which inhibits Calcineurin mediated dephosphorylation of CREB, leading to CREB mediated transcriptional upregulation of IFNγ and perforin. ‘’ indicates the steps inhibited by zinc.

Multiple studies have investigated the effect of zinc on T cells, which has been elegantly reviewed by Kim and Woo Lee (Kim et al., 2021). In brief, intracellular Zn^2+^ level is high in activated T cells. ZIP6 and ZIP8 are the predominant zinc transporters present in T cells. Upon TCR stimulation, ZIP6 mediates Zn^2+^ influx and loss of ZIP6 impairs T cell activation (Colomar-Carando et al., 2019). Subsequently it was found that Src and/or Syk family kinase ZAP70 mediated phosphorylation of ZIP6 was essential for localization of the latter to the immunological synapse and Zn^2+^ influx upon TCR stimulation (Kim et al., 2021). Zn^2+^ influx also inhibits SHP-1 mediated dephosphorylation of the LCK, enabling increased phosphorylation of ZAP70 kinase by LCK at the immunological synapse (Chiang and Sefton, 2001; Štefanová et al., 2003; Figure 6). Note that Zn^2+^ also facilitates the binding of LCK to CD4 and CD8α, which is important for TCR signaling (Lin et al., 1998).

On the other hand, ZIP8 is predominantly localized to the lysosome and it mobilizes lysosomal Zn^2+^ to cytoplasm. ZIP8 expression is increased upon TCR stimulation, leading to increased translocation of Zn^2+^ from lysosome to cytoplasm, which inhibits the phosphatase activity of Calcineurin. This leads to CREB (cyclic AMP response element-binding protein) mediated transcriptional upregulation of IFNγ and perforin gene expression, which are key antiviral effectors of the host (Aydemir et al., 2009; Figure 6). Besides, Zn^2+^ is also known to modulate the activity of ERK, PI3K and STAT3 signaling pathways in T cells, thereby influencing production of proinflammatory cytokines and differentiation of Th17 cells (Kaltenberg et al., 2010; Kitabayashi et al., 2010; Plum et al., 2014).

#### 5.2.3. Zinc signaling in other immune cells

Zinc deficiency reduces natural killer cell cytotoxic activity and impairs host immune response against foreign pathogens, while zinc supplementation can reverse this effect (Fernandes et al., 1979; Fraker et al., 1982). Role of ZnTs in mast cell activation and mast cell mediated allergic reaction has been reported, indicating that ZnT5 mediates FcεRI signaling which leads to activation and translocation of PKC to plasma membrane. PKC stimulates nuclear translocation of NF-κB and cytokine production in mast cells (Nishida et al., 2009). Zinc has also been shown to increase the production of IFNα in leucocytes (Samad et al., 2021). Zinc transporters ZIP7 and ZIP 10 are essential for B cell development and BCR-induced B cell proliferation (Hojyo et al., 2014; Anzilotti et al., 2019).

## 6. Possible mechanism (s) controlling the antiviral function of zinc in hepatitis viruses

Exact molecular mechanism of antiviral function of zinc against any particular hepatitis virus has not been conclusively demonstrated. Nevertheless, information obtained from several independent studies strongly support the benefit of zinc action against HAV, HBV, HCV, HEV and provide a scientific basis for further investigation. Here, we attempt to extrapolate the possible mechanism (s) (based on information obtained from studies on direct and/or indirect antiviral effect of zinc on all viruses), which enable zinc to antagonize HAV, HBV, HCV and HEV infection and suggest future directions for experimental validation of those possibilities. It is important to obtain clear mechanistic understanding of zinc action in order to harness its complete therapeutic potential for management of viral hepatitis.

As mentioned in previous sections, antiviral effect of zinc against HCV has been evaluated in multiple clinical trials, which provides an overall conclusion that zinc supplementation have therapeutic benefit when taken along with standard antiviral therapy. *In vitro*, zinc shows potent inhibitory effect on HCV RdRp, although its mechanism of action remains to be explored (Yuasa et al., 2006; Gupta et al., 2022). Recently, our laboratory showed antiviral activity of ZnO nanoparticles [ZnO(NP)] and tetrapods [ZnO(TP)] against genotype 3a-HCV replicon (Gupta et al., 2022). It is possible that zinc acts by chelating magnesium ions, which is required for HCV RdRp activity or acts through other steps in controlling RdRp function (Ouirane et al., 2019).

Zinc was also shown to prevent with IFN-λ3 binding to its receptor IFNLR1, resulting in inhibition of IFN-λ3 signaling, consequently leading to an increase in HCV replication. Single nucleotide polymorphisms rs12979860 and rs809917 in IFNL gene locus clears HCV as well as inflammation and fibrosis progression in viral and non-viral liver disease (Read et al., 2017). Further studies on HCV patients with rs12979860 and rs809917 SNPs should clarify the therapeutic benefit of zinc supplementation in HCV patients.

Previous studies done in our laboratory showed the antiviral activity of zinc against genotypes 1 (g1) and 3 (g3) of HEV, which are major cause of HEV-induced hepatitis in human. Zinc inhibits the activity of HEV RNA-dependent RNA polymerase (RdRp), *in vitro* (Kaushik et al., 2017). Similar antiviral activity was also observed in cell-based models of g1- and g3-HEV, upon treatment with ZnO nanoparticles [ZnO(NP)] and tetrapods [ZnO(TP)] (Gupta et al., 2022). In agreement with the *in vitro* data, a recent report demonstrated the anti-HEV activity of zinc in ribavirin nonresponsive HEV patients (Horvatits et al., 2023).

A recent report suggests that the anti-HEV activity of zinc is mediated by its effect on host. Overexpression of Zinc-finger antiviral protein (ZAP), an interferon (IFN)-stimulated gene, inhibits HEV replication, while its knockdown by RNA interference significantly increases HEV RNA level. Silencing of ZAP also decreases interferon regulatory factor 3 (IRF3) phosphorylation in HEV infected cells. Thus, ZAP is an anti-HEV host factor, which blocks viral replication in cooperation with IFN-β (Yu et al., 2021).

An analysis of the protein–protein interactions between human and HEV proteins revealed enrichment of proteins linked to the mitochondrial oxidative phosphorylation pathway (Chandru et al., 2018). It is known that zinc wave results in production of the mitochondrial reactive oxygen species (ROS; Slepchenko et al., 2016). Future study should clarify the involvement, if any, of mitochondrial oxidative phosphorylation pathway in modulating the anti-HEV activity of zinc. Additionally, it has been shown that HEV encoded proteins have an impact on a number of cellular pathways, which may be crucial for survival of the virus inside infected cells (Wißing et al., 2021). Zinc may exert its antiviral effects by interfering with the interplay between HEV and the host signaling pathways.

HEV infection also triggers neuronal disorders such as neurological amyotrophy and Guillain-Barrre syndrome (Jha et al., 2021). HEV infects neuronal cells in culture (Drave et al., 2016). Zinc acts as a neurotransmitter, modulates intracellular and extracellular signaling, which is essential for maintaining normal neuronal physiology (Frederickson et al., 2000). Variations in intracellular level of free zinc decide between survival or death of neurons during ischemic injury (Aras and Aizenman, 2011; Slepchenko et al., 2017). Thus, zinc supplementation might act in a different manner in HEV infected neurons than hepatocytes.

Two studies reported inhibition of HAV replication upon treatment with zinc sulphate and zinc chloride (Ogawa et al., 2019; Kanda et al., 2020). Zinc chloride showed more potent anti-HAV effect than zinc sulphate. It enhanced the antiviral effect of interferon-alpha-2a against HAV (Kanda et al., 2020). Level of mitogen-activated protein kinase 12 (MAPK12) upregulated and six related genes baculoviral IAP repeat containing 3 (BIRC3), interleukin 1 beta (IL1β), proline-serine–threonine phosphatase interacting protein 1 (PSTPIP1), prostaglandin-endoperoxide synthase 2 (PTGS2), PYD and CARD domain containing (PYCARD), and tumor necrosis factor alpha (TNFα) were downregulated in zinc chloride treated cells (Kanda et al., 2020). Further investigations are warranted to uncover the possible direct/indirect role of zinc against HAV.

Limited information exists regarding direct antiviral effect of zinc against HBV to draw any mechanistic insight. One clinical trial evaluated the therapeutic benefit of zinc supplementation in HBV patients, indicating some improvement in liver function (Hayashi et al., 2007; Himoto et al., 2007). It is noteworthy that zinc binding is essential for function of the HBX protein, which is a key protein encoded by HBV (Ramakrishnan et al., 2019). Further, many host antiviral proteins and ISGs inhibit HBV replication either by targeting protein viral proteins and/or RNA. For example, host antiviral factor ZAP inhibits HBV replication (Mao et al., 2013). ISG20 selectively degrades HBV RNA (Liu et al., 2017; Imam et al., 2020). Myeloid differentiation primary response 88 (MYD88) inhibits HBV replication by promoting viral pregenomic RNA degradation and retention of viral preS/S RNA in the nucleus (Li et al., 2010). Myxovirus Resistance Gene A (MxA), an ISG, interacts with viral core protein and inhibits HBV replication (Gordien et al., 2001; Li et al., 2012). Since zinc is known to influence the activity of these genes/pathways under different conditions, additional studies involving suitable model systems are required to assess the possible therapeutic benefit of zinc in HBV cases. Knowledge obtained from our understanding of the mechanism of zinc action in other viruses should be the guiding criteria for designing experiments in HBV cases.

## 7. Challenges associated with zinc supplementation therapy

### 7.1. Rigorous cellular zinc homeostasis

Zinc homeostasis in the cell is a multistep process constituting three main stages, namely the zinc influx into the cells, zinc efflux from the cells and the storage within the cells. As described earlier, this process is realized through zinc transporters and the zinc binding proteins. In normal conditions, zinc homeostasis is tightly and neatly controlled, but while considering zinc supplementation, the dosage and the amount need to be judiciously and carefully curated. Any imbalance of zinc levels due to supplementation might cause disruption of zinc transport, leading to zinc accumulation in the cells causing toxicity. One of the major challenges to zinc supplementation is that zinc is needed in a large number of cellular processes and therefore interacts with a large number of proteins. It is really difficult to keep track of the effect of zinc supplementation on all these proteins.

### 7.2. Expression of zinc transporters in target cells and their effects

A number of reports suggest that differential expression and activity of ZIP and ZnTs are responsible for the pathogenesis and progression of chronic diseases. ZIP transporters are known to promote the invasive activity of cancers. Reduced expression in ZIPs has been seen in prostate cancer, where the cellular zinc is reduced, which accelerates the disease progression. ZIP4 is known to get atypically overexpressed in case of “acrodermatitis enteropathica.” ZIPs are known to play roles in epithelial–mesenchymal transition, anoikis resistance, and metastasis. In such cases, abnormal expression of ZIPs elevates the cytosolic zinc levels, which may extend the zinc dependent growth factor signaling and cause aberrant activation of cellular signaling pathways (Kagara et al., 2007). A myriad of secretory and membrane-bound enzymes which need zinc like matrix metalloproteinases are involved in cancer metastasis, and capture zinc in the early secretory pathway. Thus, ZnT transporters like ZnT5, ZnT6, and ZnT7 might be facilitating zinc uptake by these enzymes and their activation (Kambe, 2012). These examples merely illustrate that abnormal dysregulation of the zinc transporters have been observed in various disease and zinc supplementation could have a similar effect on these transporters and could lead to deleterious effects if not administered correctly.

Zinc interferes with the copper absorption in the body, even at a very little dose above the RDA (Recommended Dietary Allowance). Actually, higher concentration of zinc induces more expression of metallothioneins in the lumen to absorb more amount of zinc. However, this protein also has a high affinity for copper, resulting in a copper deficiency, and may cause anemia, neutropenia and myelopathy (Francis et al., 2022).

### 7.3. Zinc toxicity

Zinc is known to have many beneficial functions and has seen to play a role in protection in various infectious diseases, but excess zinc can cause zinc toxicity or side effects. Most often the zinc toxicity depends on the route of exposure to zinc or on the dosage. Zinc toxicity can be divided into two types- (a) acute and (b) chronic toxicity. Acute toxicity is caused by ingestion of salts of zinc such as zinc sulphate and zinc chloride which manifests into gastrointestinal symptoms like diarrhea, renal injury, acute respiratory distress syndrome (ARDS), liver necrosis, thrombocytopenia, coagulopathy and might lead to death in extreme cases (Barceloux, 1999). Chronic toxicity causes bone marrow damage and neurological symptoms. One of the major causes of concern in case of chronic toxicity is that if zinc is chronically ingested it can result in copper deficiency can lead to sideroblastic anemia, granulocytopenia, and myelodysplastic syndrome (Irving et al., 2003; Sheqwara and Alkhatib, 2013). Orally consumed zinc is absorbed by the jejunum of the small intestine. This process is facilitated by the metallothionein complex in the enterocyte villi (Ruttkay-Nedecky et al., 2013). Zinc binds to the metallothionine, which is known to be involved in the regulation of other metals as well, especially copper having the highest affinity to it (Ruttkay-Nedecky et al., 2013). To remove the excess zinc, the human body produces more metallothionein to prevent excess free zinc but in turn also decreases copper levels, thus, forming a dynamic antagonistic relationship. Here, the zinc homeostasis can only be maintained by the excretion of the metallothionein-zinc complex via bile and faeces. In case of zinc overdose, excretion of zinc will not be fast enough and thus it will get accumulated and cause clinical complications (Agnew and Slesinger, 2020).

## 8. Advances in zinc supplementation strategy and future perspectives

### 8.1. Zinc derivatives

Due to the advancement in nanotechnology, biomedical nanoparticles have gained considerable attention due to their prominence in biomedical applications and are being explored for molecular diagnostics, drug delivery, gene therapy. In case of Zinc based nanotechnology, zinc oxide-based nanoparticles have taken the center stage (Zhang and Xiong, 2015; Bashandy et al., 2018).

Zinc oxide nanoparticles (ZnO NPs) are important and are used in a variety of fields due to their unusual properties. ZnO nanoparticles have a small particle size which helps in the easier absorption of zinc by the body and are used as food additives and the US FDA has classified it as a GRAS (Jiang et al., 2018). In addition to this ZnO NPs are relatively inexpensive and less toxic. These properties make ZnOs an excellent candidate for biomedical applications. ZnOs have been used in anti-cancer treatment, drug delivery, antibacterial, diabetes treatment, anti-inflammation, wound healing and bioimaging (Zhang and Xiong, 2015; Mishra et al., 2017). In recent years nanoparticles of gold and silver have been predominantly studied and deployed against viral diseases. However, Zn nanoparticles might be relatively less toxic than silver and gold nanoparticles and more cost efficient. ZnO nanoparticles have proven to be successful as a therapeutic strategy for various viral diseases, although they have not been very extensively studied in animal as well as human models, but promising results have been obtained in various cell-based models (Aderibigbe, 2017). Zinc oxide nanoparticles have been shown to have a virostatic effect against HSV-1 and were able to efficiently trap virions from entering the human corneal fibroblasts (Mishra et al., 2011). Surface modified zinc oxide nanoparticles have been shown to alter the infection of Herpes simplex virus -I by neutralizing the virus by utilizing the electrostatic interference caused by the hydrophobic zinc nanoparticles (Farouk and Shebl, 2018). PEGylated zinc oxide nanoparticles have been shown to be successful in inhibiting H1N1 viral infection and there was a 1.2 log10 TCID50 decrease in the viral titer (Ghaffari et al., 2019). Further ZnO nanoparticles suppressed replication in case of nidovirus and also have been shown to disrupt the replication of a range of RNA viruses (Gurunathan et al., 2020). In MA104 cells (African green monkey fetal kidney), ZnO nanoparticles led to a 10-fold decrease in the chikungunya viral load (Kumar et al., 2018).

In addition to this, ZnO tetrapods have also been used as an alternative for the traditional nanoparticles (Mishra and Adelung, 2018). The unique 3D structure gives them a higher flexibility as a biomedical engineering tool than traditional spherical or 1D nanoparticles as tetrapods avoid the agglomeration issues faced by the other two. The first use of ZnO nano tetrapods was for the efficient delivery of plasmid DNA (Nie et al., 2006). Furthermore, zinc tetrapods have been used to trap virus particles using oxygen vacancies on the synthesized ZnO tetrapods. Polar surfaces were created in the tetrapods due to the oxide, which helped in trapping the negatively charged functional glycoprotein groups present on the virion surface (Mishra et al., 2011). Dendritic cells could easily take up such entrapped virus particles and neutralize them. This success of this strategy has been shown in humans and mice in the cases of HPV, ZIKA, HIV and Dengue. Apart from this ZnO nanoparticles and tetrapods have been shown to have an antiviral role in HSV, HEV and HCV by suppressing the viral replication (Mishra et al., 2011; Gupta et al., 2022).

Although zinc based nanoparticles have been shown to play antiviral roles they need to be further characterized to be successfully established as a commercially available therapeutic for viral diseases.

### 8.2. Modulators of zinc transporter

Zinc transport can be modulated by zinc ionophores. Zinc ionophores have been shown to inhibit replication of many viruses *in vitro*. Four zinc ionophores have shown to have antiviral roles which are Pyrithione, Hinokitiol, PDTC and Chloroquine. Pyrithione, Hinokitiol and PDTC inhibit the replication of the following viruses *in vitro*: coxsackievirus, equine arteritis virus, SARS-CoV, HSV, mengovirus and rhinovirus (Lanke et al., 2007; Krenn et al., 2009; Te Velthuis et al., 2010; Qiu et al., 2013). Chloroquine inhibits the replication of the following viruses: HCV, HCoV-229E, MERS-CoV, SARS-CoV, HIV-1 and Zika virus (Romanelli et al., 2004; Delvecchio et al., 2016; Read et al., 2017; Chandru et al., 2018). In addition to this, another zinc ionophore, Quercetin is known to inhibit replication of Influenza A virus and Rhino virus (Wu et al., 2016; Mehrbod et al., 2021). These studies indicate that zinc ionophores may be used as antivirals and since zinc has a significant role to play in liver homeostasis, the use of these ionophores is likely be an excellent antiviral strategy against hepatitis causing viruses.

### 8.3. Future perspectives

ZnO NPs have shown promise in biomedical applications due to their anti-viral, anti-bacterial and anti-diabetic roles. Inherently toxic, the ZnO NPs inhibit cancerous cells as well as bacteria by generating intracellular ROS generation. This activates the apoptotic signaling pathway making these nanoparticles excellent anticancer and anti-bacterial agents. Furthermore, Zinc nanoparticles and tetrapods have shown significant antiviral activities ranging from affecting replication, to neutralizing viruses and influencing viral entry. As drug carriers, ZnO NPs enhance therapeutic efficiency by promoting the bioavailability of the drugs or biomolecules.

The FDA has listed ZnO NPs as a safe substance. However, they should be further explored and studied as there are no comparative analysis of the biological advantages of Zinc nanoparticles over other metal nanoparticles. Further, there is lack of information on evidence based randomized trials and animal studies emphasizing their therapeutic roles. Focused studies on these aspects would help us better understand their diagnostic and therapeutic potential.

It is also important to be cautious and careful while interpreting and extrapolating the data obtained from cell-based experiments to patient studies. Generally zinc compounds are used in micromolar (μM)-millimolar (mM) concentration in the cell-based studies to evaluate their antiviral potential whereas plasma zinc concentration ranges between 10 and 18 μM in human (Rükgauer et al., 1997). On top of that free zinc levels are very tightly controlled *in vivo*. Although intracellular level of zinc maybe in micromolar concentration, most of it is bound to metallothioneins, thereby reducing the free zinc concentration to nanomolar scale (Krężel and Maret, 2006; Zheng et al., 2017). Such complex regulation makes it difficult to fully harness the therapeutic benefit of zinc based on the data obtained in cell-based experiments. Therefore, even though a zinc compound may exhibit very potent antiviral effect in laboratory condition, it needs stringent evaluation in the clinic.

The treatment of viral hepatitis has evolved rapidly over the last few years especially with the introduction of curated therapies for Hepatitis C. In addition to this the improvement in HAV, HBV and HEV vaccination has also led to a significant advance in viral hepatitis management. Viral hepatitis treatment is still dependent on a few drugs like Ribavirin and PEGylated Interferon but none of these are known to specifically target these viruses. Thus, targeted therapies can be further explored; one such strategy could be the use of zinc salt, zinc nanoparticles and zinc ionophore based therapies. We have recently shown the antiviral activity of zinc oxide nanoparticles and tetrapods against HEV and HCV, which could pave the way for the development of new therapeutic strategies against these viruses (Gupta et al., 2022).

Zinc oxide nanoparticles have shown promising antiviral effects on various viruses, but further research needs to be performed to explore and understand the underlying mechanism of zinc nanoparticles dependent antiviral activities. Zinc seems to inhibit the enzyme activities of viral protease and polymerases and is also involved in the physical processes of viral attachment and uncoating (Mishra et al., 2011). However, it is important to study these in clinical scenarios as zinc could become a viable supplement to the traditional viral treatments. A number of *in vitro* studies have demonstrated that free zinc possesses a strong antiviral effect through the trials with creams and tablets containing higher amounts of zinc (Mishra et al., 2011). These studies also tell us that when zinc is used at therapeutic doses and in the right form could improve viral clearance in case of acute and chronic infections. Zinc supplementation can be successful in two ways: it can be either be administered to improve the systemic immunity and the antiviral response in zinc deficient patients or can be used to specifically target viral replication and the symptoms of infection (Mishra et al., 2011). Although zinc-based strategies have been shown to have various roles in inhibiting the viral replication, there is a lack of information on whether these zinc-based strategies are sufficient as a standalone treatment or in enhancement of the effect of other antiviral treatments. Zinc supplementation with Ribavirin or PEGylated IFN-α reduced the side effects of Ribavirin/PEGylated IFN-α in case of HCV and HEV chronic patients but had no additive effect on these treatments (Farouk and Shebl, 2018; Suda et al., 2019). It has also been proven that in chronic patients of HCV and HBV zinc supplementation induces anti oxidative functioning of the liver preventing further liver damage during the treatment with Ribavirin and PEGylated IFN-α (Murakami et al., 2007). Zinc supplementation has also been shown to reduce inflammation and production of cytokines like NF-κB in chronic HCV and HBV patients (Nagamine et al., 2000). Accumulating data, through both *in vitro* and clinical studies, suggest that zinc supplementation in addition to other antiviral therapy is a viable treatment regimen for viral hepatitis. Although this needs to be further elucidated through large cohort based clinical studies.

## Author contributions

SK, SA, SN, CR-K, and MS wrote the manuscript. MS and CR-K edited the manuscript draft. MS conceptualized the review. All authors contributed to the article and approved the submitted version.
